# Adaptive Optics RTX1 Imaging for Early Detection of Retinal Vascular Remodeling in Hypertensive Retinopathy: A Review

**DOI:** 10.3390/jcm15093376

**Published:** 2026-04-28

**Authors:** Mateusz Zabochnicki, Agnieszka Łebek-Szatańska, Monika Łazicka-Gałecka, Anna Zaleska-Żmijewska, Andrzej Januszewicz, Jacek P. Szaflik

**Affiliations:** 1 Department of Ophthalmology, Public Ophthalmic Clinical Hospital (SPKSO), Medical University of Warsaw, Sierakowskiego 13 Street, 03-709 Warsaw, Poland; monika.lazicka-galecka@wum.edu.pl (M.Ł.-G.);; 2 Department of Hypertension, Cardinal Stefan Wyszynski Institute of Cardiology in Warsaw, Alpejska 42 Street, 04-628 Warsaw, Poland

**Keywords:** adaptive optics, RTX1, hypertensive retinopathy, hypertension, retina

## Abstract

Background/Objectives: Arterial hypertension might lead to serious organ damage and complications like hypertensive retinopathy. The retina is the only place in the human body where microscopic blood vessels can be directly investigated. This enables early diagnosis of arterial hypertension-mediated organ damage. Untreated hypertensive retinopathy leads to vision loss in its advanced stages. There are many methods of assessing changes in the arterioles; however, the most accurate is adaptive optics (RTX1™ device with AODetectArtery software, ver. 3.0., Imagine Eyes, Orsay, France). It provides a resolution of 1.6 μm, which is superior to conventional imaging techniques. Optical coherence tomography angiography can serve as an early, minimally invasive marker of microvascular damage. Across the studies analyzed, the WLR (Wall-to-Lumen Ratio) exhibited significantly higher values when comparing individuals with hypertensive retinopathy to normotensives (0.31 vs. 0.26). The main aim of this review is to present the application of adaptive optics in the early diagnosis of hypertensive retinopathy. Methods: The search strategy included 267 original studies, among which 12 were selected to be described and analyzed in this review based on criteria including original research and studies performed on humans with hypertensive retinopathy. Results: RTX1™ enables the assessment of arterial parameters such as the Wall Thickness (WT), Lumen Diameter (LD), Outer Diameter (OD), Wall-to-Lumen Ratio (WLR) and Wall Cross Sectional Area (WCSA). These parameters differ depending on the arterial hypertension. The WLR was identified to be the parameter that differs in the vast majority of analyzed studies when comparing hypertensive patients to normotensive patients. Vascular parameters were also found to change depending on different organisms’ states, treatment applications and etiological causes of disease. Furthermore, changes in retinal arterial parameters were associated with increased cardiovascular risk in observational studies. RTX1™ was also identified to provide very good intra- and interobserver variability. Conclusions: RTX1™ is a valuable tool in the examination of arterial vessels and in establishing associations between retinal vascular parameters and a patient’s clinical state.

## 1. Introduction

Hypertensive retinopathy is an eye disorder that is etiologically caused by chronic or acute elevation in blood pressure. Other, less commonly known ocular manifestations of arterial hypertension are hypertensive choroidopathy and hypertensive optic neuropathy [1].

The literature shows that the prevalence of hypertensive retinopathy among patients with arterial hypertension may range from 37% to 90% among different studies [2,3,4]. However, in some studies, it ranged from 8.3% to 15.0% of the population [5]. Studies indicated that adequate treatment of elevated blood pressure, resulting in good control of the disease, might promote the regression of retinal vascular changes, including focal narrowing [6]. Patients with detected hypertensive changes should receive intensive antihypertensive treatment in order to decrease the blood pressure level. Because of this, they might benefit from a more-intensive clinical approach.

The mechanisms that lead to hypertensive retinopathy are oxidative stress, low-grade inflammation, vasospasm, reduced production of vasodilating factors, endothelial damage and platelet activation [7,8,9].

There are two vastly used scales to visually assess hypertensive damage to the retina. The first is the Keith–Wagener–Barker (KWB) scale, and the second is the Mitchell and Wong (MW) classification. The Keith–Wagener–Barker scale includes five stages: 0—normal; 1—mild generalized retinal arteriolar narrowing; 2—more severe narrowing, focal changes, arteriovenous nipping; 3—grade 2 and retinal hemorrhages, microaneurysm, exudates, cotton wool spots; and 4—grade 3 and optic disc edema, macular edema [10]. The Mitchell and Wong classification is as follows: No retinopathy—no detectable signs; Mild—generalized or focal arterial narrowing, arteriovenous nipping, arteriolar wall opacity (silver wire); Moderate—hemorrhage, microaneurysm, cotton wool spot, exudates; and Malignant—moderate with optic disc swelling [11]. In some studies, grade 1/2 or Mild retinopathy is associated with stroke and coronary artery disease [12,13]. Other possible markers of changes present in the retinal vascular system include vessel tortuosity, which indicates twisting and curvature of the retinal blood vessels [14].

Untreated hypertensive retinopathy may present with vision disturbances or, more rarely, as vision loss. In some patients, especially those suffering from severe secondary hypertension forms signs such as headaches or photophobia, they are usually present before sight loss. Macular exudative changes and optic disc edema (grade 4) may lead to permanent visual acuity loss. However, in the early and moderate stages of hypertensive retinopathy, the patient could remain asymptomatic [15,16,17,18].

The main clinical problem is that hypertensive retinopathy is a microvascular disease. Because of this situation, in-depth analysis of the retinal vasculature is essential to effectively treat and diagnose it.

Adaptive optics (AO) was originally developed in astronomy to correct aberrations created by vibrations of the atmosphere. In addition, it enables significantly better image quality, better resolution and higher contrast [19]. Even in the normal and healthy human eye, aberrations are present and lead to a decrease in the overall performance [20]. The AO system consists of a Hartman–Schack wavefront sensor to measure aberrations coming from the examined retina, a deformable mirror divided into smaller pieces that dynamically adjust themselves to compensate for detected interferences in real-time and a control system, that coordinates all the pieces working together [21]. A deformable mirror works by changing its surface shape under electric impulses [22]. With the ability to compensate disruptions from the ocular system, AO allows investigators to achieve a resolution of around 2 μm. That level of accuracy enables visualization of single cone photoreceptors [23]. To be applied clinically, AO must be combined with other technology. The currently available options are fundus camera, scanning laser ophthalmoscopy and OCT [24]. Fundus camera AO creates fewer motion artifacts compared to AO scanning laser [25]. AO is minimally invasive, safe and very well tolerated by examined patients. It achieves a quality level that is impossible to reach with any other in vivo method [26]. The practical use of AO in ophthalmology started over 25 years ago [27]. Adaptive optics vascular parameters could be used as an applicable tool to detect and monitor altered vascular biomarkers among hypertensive patients [28]. The most important advantage of the AO is the ability to analyze vessels in a very high-resolution, that are not accessible to examine minimally invasively in any other region of the organism [29].

RTX1™ (Imagine Eyes, Orsay, France) consists of a microscope fundus camera combined with adaptive optics technology that enables a resolution of 1.6 μm using an infrared wavelength of 850 nm. It creates an analyzed retina surface area of approximately 1.2 × 1.2 mm (1200 μm × 1200 μm) in the emmetropic eye, enabled by a 4 × 4-degree field of view. Within the available examination area, the exact spot of acquisition is available to be set by the operator. The fixation point is always set by the operator of the device [30]. The RTX1™ system can compensate for spherical error between −10.0 D and + 8.0 D and up to 5.0 D of astigmatism. The user of the system can also set the depth of the image to find the eligible focus point depending on which layer or structure needs to be visualized. Focus depth ranges 600 μm [31]. The time necessary to acquire one image is approximately 4 s. In that period, RTX1™ performs a series of 40 images. After collection of the retinal images is finished, they can be assessed with dedicated programs: AODetectArtery^®^ (Imagine Eyes, Orsay, France) to analyze vessels and AODetect^®^ to analyze photoreceptors [32,33]. Because of that, RTX1™ enables quantitative, comparable analysis of measured parameters between different patients and also between measurements of the same patient in time intervals. That enhances the value of collected clinical data. This is possible because RTX1™ allows us to set exactly the same coordinates of the measurement during follow-up visit [34,35]. Additionally, in ESH guidelines, adaptive optics is highlighted as a useful tool to assess hypertension-mediated organ damage in the eye. Moreover, devices like RTX1™ could enable precise assessment of retinal arteriole structure and support early recognition of hypertensive changes [36].

After the end of the examination process, RTX1™ is capable of measuring different vascular parameters: Vascular Diameter (VD), Wall Thickness (WT), Wall-to-Lumen Ratio (WLR), Lumen Diameter (LD), Wall Cross Sectional Area (WCSA). Wall Thickness is calculated based on the measurement of the Lumen Diameter and two wall diameters, Wall-to-Lumen Ratio is calculated from the equation: 2x (WT/LD). Wall Cross Sectional Area is calculated automatically by the RTX1™ device software [37].

Arterial hypertension is a major modifiable risk factor for cardiovascular diseases [38]. According to WHO (World Health Organization), arterial hypertension is defined as systolic blood pressure of 140 mmHg or higher and/or diastolic of 90 mmHg or higher [39]. It is estimated that at least 31% of the adult population of the world has elevated blood pressure levels, and it is projected to increase. Based on WHO data, in 2019, 1.3 billion people suffered from hypertension and over 75% of these cases occurred in low- and middle-income countries [40]. The prevalence in low- and middle-income countries is rapidly increasing [41]. Two major groups based on etiology were highlighted: primary and secondary hypertension. Primary hypertension (also known as essential hypertension) with the prevalence of around 80% is defined by the absence of a single identifiable triggering factor [42]. Secondary hypertension generally results from a known external factor or illness.

Untreated hypertension causes hypertension-mediated organ damage (HMOD) in several organs, including: heart, brain, kidneys, vascular system and eyes [43,44]. The early detection of the HMOD is crucial to reduce the risk of progression and prevent more serious events from happening [45]. In the field of ophthalmology, rapid diagnosis followed by adequate treatment, even in severe cases, may help to preserve patients’ vision. It can also protect retinal cells and layers from atrophy [46].

Regarding ophthalmological implications of elevated blood pressure, arterial hypertension is a recognized risk factor for non-arteritic anterior ischemic optic neuropathy, branch retinal artery occlusion, central retinal artery occlusion, branch retinal vein occlusion, central retinal vein occlusion and retinal detachment [47,48,49].

The retina is a highly specialized type of neural tissue that originates embryologically from the forebrain. It consumes vast amounts of oxygen, energy and nutrients. Any disturbance in the retinal perfusion system leads to pathologies and diseases [50]. Therefore, it requires an efficient blood supply system.

Another available imaging techniques in hypertensive retinopathy include fundus photography, optical coherence tomography (OCT), optical coherence tomography angiography (OCTA), fluorescein angiography (FA) and adaptive optics (AO-RTX1™ device, AO scanning laser and more).

Fundus photography is a fast, accurate and economically viable method that allows comparison of changes over time between patients’ visits. Because of this, it enables better assessment of the pathological changes progression [51]. It utilizes KWB or MW grading systems to standardize the assessment. This method is limited by the physician’s experience, and does not allow insight into the changes of the tiniest retinal vessels. Classic fundus photography does not allow us to measure the exact diameters of the arterioles and veins. However, semiautomated computer systems support clinicians with arteriovenous nicking detection and vessel width calculations [52,53].

Optical coherence tomography (OCT) is a non-invasive technology that enables obtaining high-resolution images, based on interference between light reflected from an examined object and a reference signal of the local tissues. It produces a cross-sectional image of the retina, that can be analyzed [54]. OCT is a useful tool that could be applied in hypertensive retinopathy diagnostics. It might allow the measurement of vessels diameters (by calculating the distance between the reflections of the walls), and by that, assessing arteriovenous ratio. It is also repeatable, which enables good follow up in the prolonged observation [55]. OCT was primarily designed to assess changes in retinal morphology and thickness. In the Akay et al. [56] study, it was presented that there was no difference in Retinal Nerve Fiber Layer (RNFL) between hypertensive patients and healthy controls. However, Ganglion Cell Complex (GCC) decreased significantly in the hypertensive group (parameters describing morphological changes in the retinal tissue) [56]. Moreover, the mean arteriovenous ratio was significantly lower in the hypertensive group of the patients compared to the normotensive group [57].

OCTA (optical coherence tomography angiography) is a technique based on OCT, that enables a non-invasive evaluation of the retinal blood vessel density. Because of this fact, the signal of the flowing blood is variable. Analysis of the repeated scans, in the same area of the retina, could detect the presence of vessels, even achieving a resolution that allows assessment of the retinal microvasculature [58]. OCTA can also be used to analyze hypertensive changes. They can present as focal capillary sparsity, morphological disorder, lack of perfusion, macular vessel defects and microangiomas [59]. OCTA allows physicians to investigate superficial and deep vascular density in the macula region, which is decreased in hypertensive eyes [60]. Changes in the microvasculature of choriocapilaries were associated with impaired renal function. It is because changes in retinal vessels may mimic changes in the renal microvascular system [61]. OCTA presents as the superior method in terms of showing ischemic areas and Elsching spots (deep orange color spots) [62]. However, the cost of OCTA devices range from 80 000.00 USD up to 150 000.00 USD, which makes it a very costly and not very effective method of assessing exact retinal changes, when compared to RTX1 [63]. Additionally, in terms of availability, OCTA appears to be more accessible because of possible combination with OCT devices, compared to RTX1, which requires a separate device.

Fluorescein angiography (FA) allows very accurate visualization of retinal vessels, using intravenous dye injection, followed by a series of high-resolution fundus photographs. However, using fluorescein sodium as a contrast marker might lead to complications like vomiting, syncope, respiratory disturbances or even death. Additionally, performing FA in children or pregnant women should be avoided [64]. FA might demonstrate ischemic focal areas of the retina, vascular occlusions, arteriolar and morphological changes [65]. Subretinal leakage, disc leakage, vascular remodeling and delayed filling could also be detected [47,66]. Changes expressed in the fluorescein angiography differ based on the duration of the hypertensive state. They might be undetectable if the time period since the beginning of the disease is short [67]. A major advantage of the FA over OCTA is that it allows an examination of the whole retina with 75 degrees or more of the range, compared to only one section analysis [68].

Retinal arterioles were identified to present a eutrophic remodeling pattern, indicated by higher WLR and smaller LD. They can also present with a hypertrophic pattern in a prolonged hypertensive state or diabetes [69,70,71,72,73].

Artificial Intelligence (AI) is being integrated into different branches of medicine. Regarding ophthalmology, that includes the diagnosis of AMD, diabetic retinopathy, retinopathy of prematurity and other diseases. It includes machine learning of computational algorithms [74]. In Akbar et al.’s study, the deep learning model achieved over 99% accuracy in the classification and grading of hypertensive retinopathy based on photographs of the retina [75]. AI models are capable of analyzing fundus photographs and assessing the arteriovenous ratio, vessel tortuosity, grade severity of changes, detecting hypertensive lesions and assigning proper stages in classification systems [76]. To increase the accuracy of the diagnosis, approaches using hybrid architecture consisting of different mechanisms of assessing the same pictures are utilized. That introduces a self-checking factor to the AI and helps to achieve over 90% accuracy [77]. Neural networks seem to be a promising direction for future research, outperforming more classic approaches [78]. Diagnostics of the eye fundus diseases, based on deep learning models were proven to be effective, repeatable and fast, especially in hypertensive and diabetic retinopathy [79]. Moreover, some AI programs evaluating diabetic retinopathy were even approved by the FDA (Food and Drug Administration, Silver Spring, MD, USA) [80]. To further enhance detection of microvessel changes, Artificial Intelligence is being introduced into OCTA, achieving interestingly high capabilities to predict cardiovascular risk [81]. Moreover, some AI models show significantly high accuracy in assessing hypertensive retinopathy changes, even in early stages [76]. However, these hybrids are based mostly on indirect measurements like vessel density in contrast to adaptive optics RTX1. That enables direct visualization and measurement of retinal vessels. Because of that, RTX1 may serve as a complementary gold standard for microvascular phenotyping to validate AI-derived analyses.

Adaptive optics enables high-resolution quantitative assessment of microvessels and might provide detailed characterization of vascular changes unachievable with standard imaging techniques.

The aim of this review is to present possible applications and utility of the adaptive optics technology, based on the adaptive optics fundus camera RTX1™ in the diagnostic process of hypertensive retinopathy and to compare results of currently available research, utilizing adaptive optics to assess vascular hypertensive damage.

## 2. Materials and Methods

Authors incorporated elements from PRISMA-style reporting to enhance the transparency of this study. The authors searched the PubMed (National Library of Medicine, Bethesda, MD, USA) database from 1951 to the present (May 2025) to identify studies that evaluated the impact of arterial hypertension and hypertensive retinopathy on vascular parameters of the retinal and choroidal tissues. PubMed was selected due to its wide coverage of adaptive optics and ophthalmological literature. All of the analyzed studies utilized an adaptive optics fundus camera (AO-FC) to diagnose ocular conditions. The studies that were not fully written in English were removed from further analysis. The search strategy included the following terms: RTX1™ hypertension, adaptive optics hypertension, RTX1™ hypertensive retinopathy and adaptive optics hypertensive retinopathy. We have only included original articles performed on human patients suffering from arterial hypertension and presenting with hypertensive retinopathy changes in the retina. Studies were required to report at least one quantitative retinal vascular parameter. Exclusion criteria were: studies in languages other than English, studies using different adaptive optics devices, review articles or case studies. Study selection process included: identification of research, excluding duplicates, abstract reviewing, main text analysis and assessment of the used methodology.

**Figure 1:**
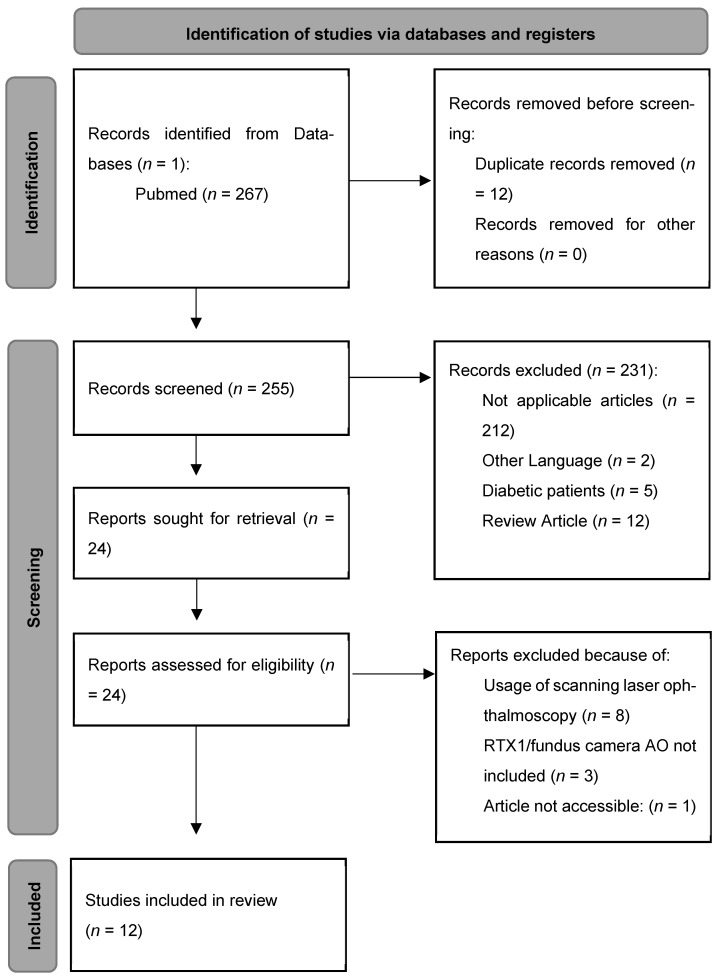
Methodology of the inclusion process (PRISMA-style flow diagram based on PRISMA templates [https://www.prisma-statement.org/prisma-2020-flow-diagram] URL (accessed on 8 April 2026).

Literature search was performed independently by two reviewers (authors) in this study. Data extraction was performed independently. Differences were resolved through discussion. The following data were extracted from each study: main characteristics, study design, population characteristics, presence of a hypertension group, measured parameters, additional data, main results. This strategy resulted in 267 studies that were identified and screened below (Figure 1). Abstracts of studies that included different matters of research, not related to RTX1™, as well as studies utilizing adaptive optics in combination with scanning laser ophthalmoscopy were excluded. Studies assessing diabetic changes were excluded. However, publications analyzing diabetes and hypertensive changes independently were evaluated in this review, focusing on the hypertensive retinopathy. A synthesis based on differences between hypertensive and normotensive patients and associations between vascular parameters was conducted. In total, 12 studies met the eligibility criteria to be fully included in this review. Because of the heterogeneity of the included studies, formal bias assessment was not performed—this is the limitation of that study. A small number of the included studies may increase the selection bias risk. The authors explain that a very limited availability of adaptive optics research evaluating hypertensive retinopathy forced inclusion of only 12 articles.

A formal risk assessment was not performed because of heterogeneity among analyzed studies. Only a qualitative assessment of methodology was applied considering sample size, study design and patient selection. The quality of the included studies was assessed as moderate. However, QUADS-2 analysis was partially performed, resulting in a moderate risk of bias in the majority of the analyzed studies. This was caused by non-random selection of the participants, highly selected patients’ populations and missing reference standards. Index testing was generally moderate because of variable protocols. The reference standard was difficult to assess, because of the relative novelty of the method. Studies were mostly cross-sectional, which excluded longitudinal follow up of the patients. Patients’ selection criteria, different sample sizes and variable study protocols might have interfered with the overall assessment of the studies.

In terms of statistical analysis, due to heterogeneity in study design and results reporting, a formal metanalysis was not performed. Because of that, a qualitative synthesis was provided. Differences in statistical methodologies were considered during interpretation. Possible bias sources, including lack of adjustments for clustering and multiple comparisons, could influence the final outcome.

## 3. Results

Analyzed studies were published between 2014 and 2024. Research was conducted on populations from France (six studies), Italy (four studies), Germany (one study) and India (one study). The adaptive optics fundus camera (RTX1™) is the only currently available commercial device with built-in AO technology. Another commonly used device is the Adaptive Optics Scanning Laser Ophthalmoscope AO (SLO-AO). It is not commercially available and requires to be specially made for the research purposes [82].

The most crucial data describing the main differences and outcomes of the studies analyzed in this review are gathered in Table 1. The data regards the first two authors, date of publication, objective, participants, gender heterogeneity stratification, vascular parameters, important additional information, subgroups, *p*-values and main conclusions. Most studies were observational, cross-sectional or longitudinal. Individual participant samples ranged from 26 to 1776 patients. In every chosen study, arterial hypertension was the leading and most influential examined factor; however, a few studies also enrolled small diabetic patients’ groups as well. Other variables included were: antihypertensive drugs, smoking, interobserver variations, different types of arterial hypertension measurements, secondary causes of arterial hypertension, body mass, age, hypertensive changes regression after treatment and more. Wall Thickness describes the thickness of the single arterial wall. Lumen Diameter (LD) combined with two Wall Thicknesses (WT) gives Outer Diameter (OD). Inner Diameter (ID) is another term for Lumen Diameter.

## 4. Discussion

Adaptive optics might enable earlier and more precise detection of microvascular damage in hypertensive patients. It provides superior insight into retinal microvasculature that could not be assessed using perfusion detecting OCTA technology.

### 4.1. Wall-to-Lumen Ratio (WLR)

Wall-to-Lumen Ratio (WLR) was the most consistently reported parameter differentiating hypertensive groups from normotensive groups. It was examined in every analyzed study of this review. WLR describes the relationship between ID and OD. Studies report a general increase in WLR values in patients suffering from arterial hypertension [70,83,85,86,87,89].

Across analyzed studies, WLR was almost consistently increased in hypertensive patients despite internal variability of different studies. It is worth mentioning that varying correlations suggest that WLR may be associated with chronic vascular remodeling.

Gallo et al. found that WLR has a very low correlation with systolic and diastolic blood pressure (r = 0.220, *p* < 0.001; r = 0.154, *p* < 0.001, respectively) [70]. Furthermore, Rosenbaum et al. also described a very poor correlation between WLR and systolic and diastolic blood pressure (r = 0.12, *p* < 0.001; r = 0.22, *p* < 0.001, respectively) [85,86]. In contrast, Koch et al. described a good correlation between WLR and systolic and diastolic blood pressure (r = 0.582, *p* < 0.01 and r = 0.559, *p* < 0.01) [83]. These differences might be caused by the fact that Koch et al. [83] examined treatment naive patients. These observations are additionally limited by different sizes of research groups.

Mehta et al. described age to be an independent factor for WLR (R^2^ = 0.049, *p* > 0.05) [89]. However, Gallo et al. described that WLR increases with age [70]. Meixner et al. adjusted vascular changes for age and found that hypertensive patients had higher age-adjusted WLR than normotensive (0.3 vs. 0.26, *p* < 0.001, respectively), the correlation coefficient for the hypertensive group was r = 0.539 (*p* < 0.01). They also found that WLR was Wall Thickness-dependent (r = 0.715, *p* < 0.01) [72]. These differences might be caused by varying study cohorts in terms of mean age and region of the world in which studies were conducted. Gallo et al. aimed to set cut-off values to distinguish hypertensive patients from the healthy group. The result was WLR > 0.313 (Specificity 71.38% and Sensitivity 57.14%) [84]. In 2016 Rosenbaum et al. published two studies in which they described WT as the parameter that increased WLR [85,86]. They found that blood pressure correlated with WLR increase independently of aging. De Ciuceis et al. found that the combination of Lercanidipine + Enalapril was statistically more advantageous in reducing WLR (0.24 ± 0.01 vs. 0.32 ± 0.04, *p* < 0.05, respectively). However, the number of patients in the study groups was extremely low (only three participants in each drug combination group) [28]. Gallo et al. found that WLR reduced significantly after standard drug therapy by 5.9% (*p* = 0.015); however, it did not in resistant hypertension patients [87]. Paini et al. discovered that WLR was poorly correlated with unattended and attended systolic blood pressure values (r = 0.28, *p* < 0.0001; r = 0.38, *p* < 0.0001, respectively) and as well with unattended and attended diastolic blood pressure (r = 0.34, *p* < 0.001; r = 0.29, *p* < 0.0001, respectively) [90]. Additionally, a weak correlation between WLR and attended or unattended mean blood pressure values was described (r = 0.35, *p* < 0.0001; r = 0.377 *p* < 0.0001, respectively).

De Ciuceis et al. also performed a study describing normotensive vs. essential hypertensives and primary aldosteronism hypertensives, but also treated vs. untreated groups and assessed the incidence of cardio-cerebrovascular events. They only described a weak correlation between higher WLR in untreated hypertensive patients and high systolic blood pressure (r = 0.38, *p* = 0.03). Moreover, the occurrence of cardiovascular events could be associated with increased WLR (*p* < 0.02) based on observational data. WLR higher than 0.28 was associated with less chance of cardiovascular event-free survival. Patients treated chronically against hypertension presented similar WLR to the normotensive group [91].

In another study, De Ciuceis et al. analyzed patients divided into lean and obese subgroups and later into hypertensive and normotensive subgroups [88]. They did not find any significant difference between hypertensive obese vs. normotensive obese and hypertensive lean vs. normotensive lean. However, they did find a difference in hypertensives obese vs. normotensive lean and hypertensive lean (0.373 ± 0.042 vs. 0.253, *p* < 0.001 ± 0.034 vs. 0.259 ± 0.037, *p* < 0.001, respectively) and between normotensive obese vs. normotensive lean and hypertensive lean (0.315 ± 0.056 vs. 0.253, *p* < 0.01 ± 0.034 vs. 0.259 ± 0.037, *p* < 0.05, respectively) [88]. That leads to the conclusion that obesity could have an impact on retinal vessels and promote vascular changes.

Generally, according to analyzed observational studies, WLR seems to be a very useful parameter for evaluation of hypertensive organ damage. Only one study found no correlation between WLR and groups of hypertensive or normotensive patients [91]. Moreover, WLR correlates poorly with attended and unattended blood pressure. However, variability in WLR varies in our review, which could be explained by different antihypertensive treatment status and selective inclusion of metabolic comorbidities and populations.

Further studies describing WLR’s potential to predict unfavorable cardiovascular events are necessary. According to Rizzoni et al., microvascular changes (like altered WLR) contribute to stroke or renal dysfunction and, because of that, cardiovascular events [92].

### 4.2. Wall Cross Sectional Area (WCSA)

Wall Cross Sectional Area is another commonly described parameter in adaptive optics evaluation of the retina. The formula used to calculate it is *π*/4 × (*OD*^2^ − LD^2^). The differences between received numerical outcomes are dependent on the exact point of the arteries in which every study measured them.

Meixner et al. described a significant correlation between the WLR and WCSA (r = 0.437; *p* < 0.01) [72]. In Gallo et al.’s study, WCSA differed between the sustained arterial hypertension group and the masked arterial hypertension group (3461.1 ± 795.0 vs. 3017.8 ± 780.4, *p* < 0.01, respectively) [84]. Rosenbaum et al. presented a correlation between WCSA and age, systolic or diastolic blood pressure (r = 0.07, *p* < 0.001; r = 0.12, *p* < 0.001; r = 0.07, *p* < 0.001, respectively), but no decrease in WCSA after initiation of the treatment, which was in counter to WLR decrease after treatment [86]. In Gallo et al.’s study, resistant hypertensive patients had a statistically significant reduction in WCSA (–12.1%) after being pharmacologically treated [87]. However, that comparison is limited because Gallo et al. examined a very small group of 26 patients and, by definition, a heterogenous resistant group. In De Ciuceis et al.’s study, obese normotensive patients had higher WCSA parameters compared to both lean normotensive and lean hypertensives patients (4874 ± 1423 vs. 3687 ± 1162, *p* < 0.05; vs. 3723 ± 1298, *p* < 0.05, respectively), which again implies that obesity is a strong factor influencing retinal arterioles [88]. Mehta et al. described that hypertensives have increased WCSA compared to healthy controls (5087.98 ± 1402.24 vs. 4180.03 ± 1029.51, *p* < 0.01, respectively) [89]. In another study, Gallo et al. only resulted in correlation between WCSA and age (r = 0.254, *p* < 0.001) [70].

No statistically significant difference was reported in other studies [28,70,72,83,85,90,91]. WCSA shows decreased consistency as a marker assessing hypertensive changes, when compared to WLR. This could be caused by WCSA greater association with vascular hypertrophy (compared to WLR and eutrophic remodeling). This finding might support a conclusion, that hypertrophy is not dominant in early stages of hypertension. Additionally, WCSA might be influenced by existing comorbidities.

### 4.3. Wall Thickness (WT)

Wall Thickness can be calculated using the formula ½ (OD-LD) or (OD-LD), depending on the study methodology. Koch et al. discovered a correlation between WT and both systolic and diastolic blood pressure (r = 0.438, *p* < 0.05 and r = 0.437, *p* < 0.05, respectively) [83]. Meixner et al. described a statistically significant difference in WT values between patients with arterial hypertension when compared to healthy controls (21.7 μm vs. 17 μm, *p* < 0.001, respectively) [72]. Gallo et al. found that WT was statistically significantly higher in the sustained arterial hypertension group, compared to the controlled, masked and white-coat hypertensive groups [84]. In the Rosenbaum et al. study, higher WT in uncontrolled hypertensive patients was found, when compared to controlled hypertensives and to normotensives (25.1 μm ± 4.3 vs. 22.1 μm ± 3.9, *p* < 0.05 vs. 22.0 μm ± 2.32, *p* < 0.05, respectively) [85]. In another study, Rosenbaum et al. described a correlation between Wall Thickness and both age and systolic blood pressure (r = 0.29, *p* < 0.01; r = 0.27, *p* < 0.01, respectively) [86]. Gallo et al. discovered that standard antihypertensive therapy reduced WT by 5.7% [87]. WT in obese normotensive was significantly higher compared to lean normotensive and lean hypertensive (14.2 μm ± 2.14 vs. 11.4 μm ± 2.29, *p* < 0.01 vs. 11.4 μm ± 2.26, *p* < 0.01, respectively) [88]. In another Gallo et al. study, WT was increased in hypertensives compared to normotensives (25.29 μm ± 4.1 vs. 23.99 μm ± 3.7, *p* = 0.003, respectively) [70]. In De Ciuceis et al.’s study, patients with a higher prevalence of cardiovascular events had greater Wall Thickness compared to the no-events group (13.9 μm ± 2.50 and 12.9 μm ± 1.67, *p* = 0.02, respectively) [91].

Other authors reported no statistical difference between hypertensive and normotensive groups regarding WT [28,83,84,89,90]. There is a huge variability in WT measurements among hypertensive patients, which makes it a less useful marker, when compared to WLR. It may be explained by the fact that WLR is a more complex parameter and WT is highly dependent on exact measurement technique, which could differ between studies.

### 4.4. Lumen Diameter (LD)

Lumen Diameter (also known as Internal Diameter) tends to be decreased in hypertensive patients. Koch et al. presented a correlation between the LD and both systolic and diastolic blood pressure (r = −0.384, *p* < 0.01; r = −0.362, *p* < 0.05, respectively), but also described that HT patients had decreased LD compared to normotensive (74 µm ± 12.6 vs. 83.5 µm ± 11.2, *p* < 0.05, respectively) [83]. A statistically significant decrease in LD in hypertensive patients was also confirmed by Gallo et al. in two different publications [70,84]. Additionally, they described that masked hypertensives had smaller LD than normotensive (*p* < 0.05) [84]. Rosenbaum et al. presented a correlation between LD and both systolic and diastolic blood pressure (r = −0.05, *p* < 0.001; r = −0.21, *p* < 0.001, respectively) [86]. Gallo et al. reported a 2.3% Lumen Diameter increase after standard antihypertensive therapy [87]. No statistical difference was reported in other authors’ studies [28,72,85,88,89,90,91]. LD is another parameter that differs between studies. It might be caused by measurement techniques and vasodilatation mechanisms caused by antihypertensive treatment.

### 4.5. Outer Diameter (OD)

Outer Diameter (also known as Vessel Diameter) describes the distance between the external borders of the retinal vessel. No statistically significant difference was reported in research conducted by other authors: [28,70,83,84,85,86,87,88,89,90,91].

Only one study by Meixner et al. described a correlation between Outer Diameter and arterio-venous ratio (in the group of hypertensive and normotensive patients combined) (r = 0.297, *p* = 0.028) [72]. These results might be caused by the fact that vascular changes influence mostly walls and lumen dependencies without affecting overall vessel size.

### 4.6. Cardiovascular Risk

Cardiovascular risk assessment can be supported by the examination of retinal arterioles. AO parameters could also be associated with the risk of local complications in the retinal tissue as well as general unfavorable events [93]. However, this evidence is limited to the observational data. Mild hypertensive retinopathy was associated with a higher risk of cardiovascular diseases (Risk Ratio: 1.13) and coronary heart disease (Risk Ratio: 1.17) compared to healthy controls. Moderate hypertensive retinopathy was associated with a greater risk of cardiovascular disease than mild retinopathy (Risk Ratio: 1.25 vs. 1.13, respectively) [94]. Increased WLR value was observed in the group of patients with heart failure with preserved ejection fraction when compared to healthy controls (0.34 vs. 0.27, *p* = 0.01, respectively) [95]. Seidelmann et al. stated that narrower retinal arterioles increased the long-term risk of mortality and cardiovascular events in both genders [96]. Harazny et al. described the correlation between cerebrovascular events and higher WLR; furthermore, in the same study, poor blood pressure control correlated with increased WLR [97].

### 4.7. Healthy Population

Kortuem et al. aimed to establish healthy population vascular parameters as a reference for further research assessing pathology. They described WLR to be 0.164 ± 0.019 with no significant age, gender or intraocular pressure correlation. They also did not find any correlation between blood pressure parameters and WLR [98]. This could be explained by the limited effect of normal blood pressure on the pathological remodeling process affecting retinal arterioles. In Mehta et al.’s study, healthy controls presented with 0.23 ± 0.03 WLR and 4180.03 ± 1029.51 WCSA, respectively [89]. Another study reported significant WLR variations in young normotensive patients (31 ± 8%) (measured directly around the optic nerve) [99]. Gallo et al. examined 1500 participants and proposed a cut-off point greater than 0.31 for WLR to discriminate hypertensives from normotensive and for LD less than 78.2 as indicative of masked arterial hypertension versus normotensive [84]. These results show a lack of a standardized reference cut-off value of WLR in a healthy population.

### 4.8. Interobserver and Intraobserver Variability

RTX1™ evaluation of the retina is a researcher-based examination. The exact outcome could be different depending on the physician. However, RTX1™ is rather known to have very good intraobserver and interobserver consistency. Intraobserver variability was 6% ± 0.9 in the single wall assessment and 2% ± 0.2 in the wall + lumen assessment [72]. In De Ciuceis et al.’s study, interobserver and intraobserver variability was also estimated as much better in AO compared to scanning laser Doppler flowmetry [88]. In Mehta et al.’s study, consistency between researchers regarding WCSA and WLR was 0.97 and 0.96 [89]. Another study found that intraobserver variability was 2.2 ± 1.9 and interobserver variability was 1.6 ± 1.8, which stays in consistency with previously described studies [35].

### 4.9. Factors Influencing Retinal Arterioles

Attended and unattended blood pressure measurements provide similar correlation between blood pressure values and vascular changes. Attended means that measurement is taken by a healthcare professional, while unattended refers to measurement taken by the patient himself, often in the home environment [100]. The measurements of blood pressure are environment-dependent. They provide lower outcomes when measured in unattended conditions compared to attended. However, only the WLR parameter correlates positively in a similar way with both types of blood pressure [90].

Primary aldosteronism (PA) is a known cause of secondary hypertension, which results in arterial hypertension-mediated organ damage [101]. It was shown by Rosei C. et al. that PA causes a greater increase in the WLR, when compared to essential hypertension (0.31 ± 0.03 vs. 0.27 ± 0.04; *p* = 0.01, respectively) [102].

The state of pregnancy is able to alter vessels morphology. For instance, WLR fluctuations before and after postpartum could be associated with the vascular system adaptation process [15].

Vascular parameters can change before and after an endurance exercise session. It is caused by increased blood pressure leading to vasoconstriction. Statistically significant reductions in OD (*p* = 0.047), WT (*p* = 0.017), WLR (*p* = 0.046) and WCSA (*p* = 0.016) were described by Żmijewska et al. in the group of healthy volunteers [103].

Current smoking was described as a significant factor differentiating changes only in the masked arterial hypertension group vs. the sustained hypertension group (*p* < 0.001) [84]. However, in other studies no difference regarding smoking was presented [70,91].

Carstensen et al. performed a study on a monozygotic and dizygotic twins cohort; they reported an association of age and blood pressure with a higher WLR rather than hereditary component [104]. Study findings support the hypothesis that mostly environmental factors influence WLR over genetics.

Malignant hypertensive patients in the population of 27 presented a WLR of 0.39 (0.31–0.47) and a correlation between systolic blood pressure and mean blood pressure [105].

WLR was identified to correlate significantly with age by many authors [72,99]. In Harazny et al.’s study, WLR of retinal arterioles correlated significantly with patients’ age (r = 0.198; *p* = 0.001) [97].

RTX1™ can also be used to assess diabetic patients, monitoring diabetic retinopathy or its proliferative state [106]. Kupis et al. observed higher Wall Thickness, increased Wall-to-Lumen Ratio and greater Wall Cross Sectional Area in diabetic patients compared to the healthy group. This implies that diabetes is a significant factor altering retinal vascular parameters [107].

Changes in the retinal vasculature were observed to withdraw after application of adequate treatment [28,87]. The systolic blood pressure decreased by 29.3 ± 17.3 mmHg and diastolic blood pressure decreased by 14.4 ± 10, occurring together with WLR parameter decrease [86].

Included studies possessed some limitations. They differ in design—some of them were cross-sectional and others were observational studies. Secondly, sample sizes ranged from only a few to more than 1500 participants, which influences the strength of the compared results. Moreover, confounding factors like age, cholesterol levels, gender, ethnic group, detailed comorbidities, weight or Body Mass Index and drug consumption were not equally checked or described in analyses performed in the compared studies. Because of that, the results of our study should be interpreted cautiously.

## 5. Future Directions

Future research should focus on setting exact cut-off points to distinguish between pathological retinal states, which could intensify the process of applying adaptive optics fundus camera in everyday clinical practice. One of the proposed cut-off values described in this review is 0.28, to distinguish between increased cardiovascular risk [91]. Exploiting Artificial Intelligence (AI) in retinal vessels diagnostics could allow researchers to obtain more accurate outcomes, by carrying out vast amounts of diameter analyses. Additionally, assessing cardiovascular risk and vision loss risk by RTX1™ requires further research to discover exact dependencies. Auxiliary diagnostics of different diseases like obesity or genetic disorders performed with AO might be an interesting direction for future research. In-depth analysis of less common hypertensive states and establishing the exact impact of different etiological forms of arterial hypertension in terms of retinal vascular parameters require more clinical research. Especially forms like malignant hypertension, hyperaldosteronism-induced hypertension or -resistant hypertension are promising in the field for future studies. Based on ESC (European Society of Cardiology) guidelines, fundoscopy is a crucial element in assessing hypertension-mediated organ damage. RTX1 could be incorporated in that process to further enhance and/or confirm the presence of pathological damage in retinal arterioles. We hope that in the future RTX1 will be incorporated into recommended protocols.

In the future, RTX1 might be combined with Artificial Intelligence image analysis to enable personalized hypertension risk stratification assessment, beyond standard commonly used methods and algorithms. That approach would significantly increase the clinical application of RTX1, especially with AI enhancement that could reach over 90% accuracy. Another potentially interesting niche is an assessment of prospective, randomized control trials focusing on the relationship between pharmaceuticals and arterial parameters changes during and after therapy. That could enable higher personalization of introduced treatments. That kind of approach would support individualization of medicine in terms of choosing the exact moment when to treat and how to treat. Strong emphasis should be placed on high-risk populations like patients suffering from primary aldosteronism (it was confirmed that this group of patients presents with higher WLR, when compared to primary hypertension), resistant hypertension or patients with existing significant comorbidities like diabetes.

## 6. Conclusions

Adaptive optics technology (RTX1™) appears to be a very accurate tool to assess vascular morphology changes in different hypertensive states. Wall-to-Lumen Ratio differs in almost every analyzed study between hypertensive and normotensive patients. However, it is limited by a high variability between analyzed studies. Vascular parameters differ depending on the disease. AO could also be used to establish the relative risk of complications and to assess the incidence of unfavorable cardiovascular incidents. However, to fully establish its role, prospective investigations are necessary. Results received from the same patient by different researchers and by the same researcher during follow up present great diagnostic repeatability. RTX1™ could possibly be applied to monitor retinal changes in diseases like heart failure, diabetes, primary aldosteronism, obesity, AMD and more. However, there is a need for further research fully establishing vascular changes in those pathological states. Furthermore, AO might be very useful in assessing retinal vascular morphological changes, including conditions like pregnancy or malignant hypertension. Additionally, observed improvements in retinal vascular parameters in patients with controlled blood pressure are based on limited evidence presenting with heterogeneity and would benefit from large group prospective studies.

Taken together, current evidence confirms that WLR is consistently higher in hypertensive groups. However, its role in predicting long-term outcomes remains uncertain and requires further investigation.

### Limitations

Associations between vascular parameters are based on the available studies, which are mostly observational. Study groups present heterogeneity in terms of examined population size (small in Mehta et al. vs. huge in Rosenbaum et al. [86,89]), age distribution, male-to-female proportions, research methods (mostly observational, but in Gallo et al., also interventional [87]), included comorbidities (mostly no diabetes vs. diabetes included in Gallo et al. [84]; additionally, some authors did not analyze cholesterol levels), definitions (hypertension could be defined in terms of WHO or ESC/ESH standards) and antihypertensive treatment status (variable in different authors). Because of that, results’ interpretation should be made with caution. The smallest analyzed study groups included only six patients. Another important limitation is that, unfortunately, all of the analyzed studies included only patients of European or Indian provenance; therefore, a huge gap in data from other populations exists. This may introduce ethnic bias and limited applicability into other demographic groups. We are recommending the creation of a multicenter study that would involve additional populations like Asian, American, African and more to enhance generalizability and validity.

Another significant limitation is the lack of Axial Length correction performed by the authors of publications, which could possibly interfere with received results. Additionally, there is a possibility of the existence of unpublished data, that might be different in terms of relationships from analyzed studies. Currently, no available studies have presented that clinical decisions regarding patients’ treatment were made. Moreover, RTX1 outcomes can be operator-dependent and image-quality-dependent.

## Figures and Tables

**Table 1:** Main characteristics and results of the analyzed studies.

Authors	Date [Year]	Objective	Participants Normotensive to Hypertensive [N:HT]	Gender Normotensive [N] and Hypertensive [HT] [Male:Female]	Measured Parameters	Additional Information	Subgroups	*p*-Values of Main Objective	Main Conclusions
Koch E., Rosenbaum D. et al. [83]	2014	Normotensive vs. Hypertensive	30:19	N 15:15 HT 11:8	WT, LD, WLR, WCSA	All of the participants were treatment naive.	Treatment naive hypertensives	<0.01	Higher WLR in HT. Narrower LD in HT.
Meixner E. and Michelson G. [72]	2015	Normotensive vs. Hypertensive vs. Hypercholesterolemia. All parameters assessed in respect to age subgroups	36:11	21:26 Non-detailed gender stratification	WT, LD, OD, WLR, WCSA	Interobserver variation assessed. In HT patients eutrophic vascular remodeling was 5× more common.	Age-adjusted subgroups	<0.01 correlation	Higher WLR in HT.
Gallo A., Mattina A. et al. [84]	2016	Normotensive vs. Hypertensive	1434:342	N 731:769 HT 182:160	WT, LD, WLR, WCSA	To the primary 1500 patients, four HT subgroups consisting of 276 patients have been added later.	Masked and sustained subgroups	<0.001	Higher WLR in HT. Narrower LD in HT.
Rosenbaum D., Kachenoura N. et al. [85]	2016	Normotensive vs. Hypertensive	23:57	42:38 Non-detailed gender stratification	WT, LD, WLR, WCSA	Hypertensives divided into Controlled and Uncontrolled	Controlled and uncontrolled subgroups	<0.001	Higher WLR in HT.
Rosenbaum D., Mattina A. et al. [86]	2016	Normotensive vs. Hypertensive	387:613	514:486 Non-detailed gender stratification	WT, LD, WLR, WCSA	Patients with Diabetes were involved.	Diabetes	<0.001	Higher WLR in HT.
De Ciuceis C., Salvetti M. et al. [28]	2017	Only Hypertensive Lercanidipine + Hydrochlorothiazide vs. Lercanidipine + Enalapril	0:30 (only 6 examined by AO)	HT 22:8 Non-detailed AO group gender stratification	WT, OD, LD, WLR, WCSA	Only 6 patients examined with AO. Assessed results of drug treatment.	ACEI and diuretic treatment	<0.05	Lower WLR after treatment.
Gallo A., Rosenbaum. et al. [87]	2017	Only Hypertensive Resistant Hypertension vs. Arterial hypertension	0:26	HT 16:10	WT, LD, WLR, WCSA	Resistant Hypertensive patients vs. previously not controlled Hypertensive patients. Patients with diabetes were involved.	Resistant hypertension	<0.015	WLR and WCSA reduction after therapy
De Ciuceis C., Rosei C. et al. [88]	2018	Normotensive vs. Hypertensive Lean vs. Obese	21:20	N 10:11 HT 9:11	WT, OD, LD, WLR, WCSA	Interobserver variation assessed.	Lean and obese subgroups	<0.01	No difference.
Mehta R., Akkali M. et al. [89]	2019	Normotensive vs. Hypertensive	110:40	N: 43:67 HT: 27:13	WT, LD, OD, WLR, WCSA	Interobserver variation assessed.	Hypertensive patients	<0.01	Higher WLR and WCSA in HT.
Gallo A., Dietenbeck T. et al. [70]	2021	Normotensive vs. Hypertensive Normoglycemic vs. Hyperglycemic	154:201	N 82:72 HT 94:107	WT, LD, WLR, WCSA	Compared with Diabetic Groups.	Glycemia assessment	<0.01	Higher WLR and W in HT.
Paini A., Rosei CA. et al. [90]	2022	Normotensive vs. Hypertensive Attended vs. Unattended Blood Pressure Measurements	57:85	74:68 Non-detailed gender stratification	WT, OD, LD, WLR, WCSA	Unattended blood pressure values are lower compared to attended blood pressure values. Eleven patients were diabetic.	Unattended and attended	<0.001	Higher WLR in HT.
De Ciuceis C., Rosei C. et al. [91]	2024	Normotensive vs. Hypertensive Treated HT vs. Untreated HT	65:172	116:121 Non-detailed gender stratification	WT, OD, LD, WLR, WCSA	Patients with primary aldosteronism were involved. Patients with cardiovascular events had higher WLR.	Treated and untreated	<0.02	Higher WLR in untreated HT.

Abbreviations: OD—Outer Diameter (OD = VD—Vessel Diameter), WLR—Wall-to-Lumen Ratio, WCSA—Wall Cross Sectional Area, WT—Wall Thickness (WT = PT—Parietal Thickness), LD—Lumen Diameter (LD = ID—Inner Diameter). OD = LD(ID) + 2x WT(PT). No significant conflicts of interest were reported. No funding sources were reported.

## Data Availability

The original data presented in the study are openly available in PubMed.
